# Preparation of Foam Dressings Based on Gelatin, Hyaluronic Acid, and Carboxymethyl Chitosan Containing Fibroblast Growth Factor-7 for Dermal Regeneration

**DOI:** 10.3390/polym13193279

**Published:** 2021-09-26

**Authors:** Longhao Jin, Sun-Jung Yoon, Dae Hoon Lee, Yun Chang Pyun, Woo Youp Kim, Ju Hwa Lee, Gilson Khang, Heung Jae Chun, Dae Hyeok Yang

**Affiliations:** 1 Department of Orthopedic Surgery, Yanbian University Hospital, Yanji 133000, China; jlh0423@sina.com; 2 Department of Orthopedic Surgery, Research Institute of Clinical Medicine of Jeonbuk National University, Biomedical Research Institute of Jeonbuk National University Hospital, Jeonbuk National University Medical School, Jeonju 54896, Korea; sunjungyoon@jbnu.ac.kr; 3 Department of Bionanotechnology and Bioconvergence Engineering, Department of Polymer Nano Science and Technology, Jeonbuk National University, Jeonju 54896, Korea; ldh7149@jbnu.ac.kr (D.H.L.); letter95@jbnu.ac.kr (Y.C.P.); snail2684@jbnu.ac.kr (W.Y.K.); wnghkljh@gmail.com (J.H.L.); gskhang@jbnu.ac.kr (G.K.); 4 Department of Medical Life Sciences, College of Medicine, The Catholic University of Korea, Seoul 06591, Korea; chunhj@catholic.ac.kr; 5 Institute of Cell and Tissue Engineering, College of Medicine, The Catholic University of Korea, Seoul 06591, Korea

**Keywords:** gelatin, hyaluronic acid, carboxymethyl chitosan, fibroblast growth factor-7 (FGF-7), wound healing

## Abstract

Wound recovery close to the function of the native skin is the goal of wound healing. In this study, we prepared foam dressings (FDs; 2-GHC-FD-1–9, 5-GHC-FD-1–9, and 10-GHC-FD-1–9) composed of various concentrations of gelatin, hyaluronic acid, and carboxymethyl chitosan, which are chemically interconnected through amide bond formation, for evaluating wound healing. Tensile and cell proliferation tests showed that 2-GHC-FD-1–9 are suitable for wound dressing. For further evaluation, three types of FDs, 2-GHC-FD-1, 2-GHC-FD-4, and 2-GHC-FD-8 were chosen. The results of animal intradermal reactivity, water vapor transmission rate, and absorption rate of the three FDs indicated that 2-GHC-FD-8 is the most appropriate scaffold for wound healing. For wound healing acceleration, various concentrations of fibroblast growth factor-7 (FGF-7) was soaked in 2-GHC-FD-8 (2-GHC-FD-8/F1-6) and evaluated by using scanning electron microscopy, cell proliferation, release behavior, and in vivo animal tests. The FDs showed interconnected porous structures, increased cell proliferation until 8.0 × 10^−11^ M, controlled release with initial burst within 1 h, and sustained release for 48 h. The results of the animal test showed an appropriate concentration of FGF-7 for wound healing. In addition, 2-GHC-FD-8 is a suitable scaffold for wound healing. Therefore, we suggest that 2-GHC-FD-8/F3 is a useful wound dressing for accelerating wound healing.

## 1. Introduction

Ideal wound healing involves the prevention of secondary infections and the minimization of scarring. It occurs in a moist environment as re-epithelialization is promoted in a wet wound, followed by a reduction of scar formation [1]. Applying dressing to wounds can accelerate healthy wound healing by protecting the wound from bacteria and supporting a moist environment [2]. Several types of wound dressings have been reported, such as foams, hydrogels, hydrocolloids, and films, for improving wound healing [2]. Among these dressings, foam is a valuable tool for protecting trauma, controlling exuding wounds, and minimizing discomfort and pain [3]. In addition, foam dressing can support a moist environment, cushion wounds from additional trauma, provide thermal insulation, and simply applied and removed from the wounds [3].

Wound dressings have been developed using natural and synthetic polymers or their combinations [4]. Characteristics not observed in synthetic polymers, but observed in naturally derived polymers are, for example, high biocompatibility, similarity to the extracellular matrix, and minimal immunological reactions [5,6]. These unique properties are required for wound dressings.

Gelatin has been clinically tested for wound healing applications among natural polymers because of its hemostatic properties, good biocompatibility, and controlled biodegradability [7]. Hyaluronic acid (HA), a non-sulfated glycosaminoglycan (GAG), is a primary component of the skin extracellular matrix (ECM) and is related to inflammatory response, angiogenesis, and tissue regeneration; however, its confined cell adhesion and proliferation limit its application as a wound dressing [8]. Chitosan derivatives, such as carboxymethyl chitosan (CMCS), glycol chitosan, etc., has also been investigated as a wound dressing material because of its high biocompatibility, biodegradability, antimicrobial activity, nontoxicity, and moisturizing capacity [9,10]. These natural polymers have been widely investigated for biomedical applications [9,10,11,12,13,14,15,16].

In addition to biomaterials for wound dressings, growth factors in dressings can improve wound healing through the migration, proliferation, and differentiation of cells related to healing [17]. Fibroblast growth factor-7 (FGF-7) has essential functions during wound healing [18,19,20]; it stimulates the migration and multiplication of keratinocytes and improves re-epithelialization. However, when growth factors are exogenously applied to wounds, they have restricted clinical effectiveness due to low stability in vivo and limited absorption through the skin around wounds [17]. Therefore, biomaterials that control the efficacy of growth factors under exogenous conditions are required.

**Figure 1:**
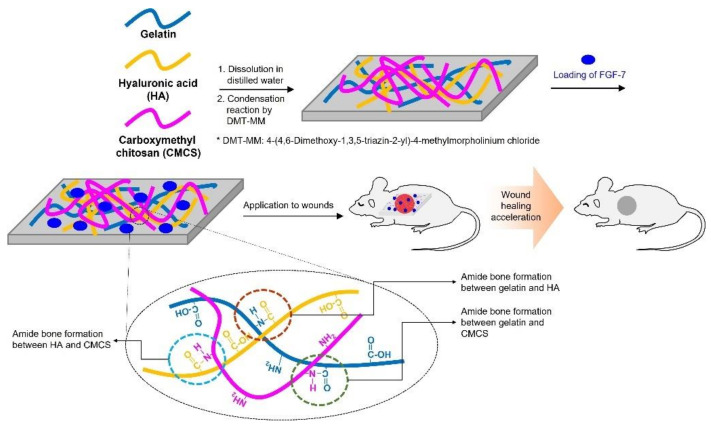
Schematic illustration of FDs made of gelatin, HA, and CMCS containing various concentrations of FGF-7 and amide bond formation among the polymers by condensation reaction. The amide bonds were formed by condensation reactions between the carboxylic group of gelatin and the amine group of HA, between the carboxylic group of HA and the amine group of gelatin, and between the carboxylic group of HA and the amine group of CMCS.

The goal of this study is to investigate the efficacy of foam dressings (FDs), composed of gelatin, HA, and CMCS containing FGF-7 on wound healing acceleration in vivo (Figure 1). To the best of my knowledge, this combination is the first study evaluating its wound healing acceleration using wound animal model; this study must be carried out for the development of advanced wound dressings. The surface morphology of the foam dressings (FDs) was characterized using scanning electron microscopy (SEM). The biocompatibility tests were evaluated by NIH/3T3 cell proliferation, intradermal reactivity, water vapor transmission rate (WVTR), and absorption rate. The incorporation of FGF-7 in FDs was investigated using Fourier transform infrared spectroscopy (FTIR). The release behavior of FGF-7 was evaluated using the Cell Counting Kit-8 (CCK-8) assay. Wound healing efficacy was evaluated using a skin incisional mouse model for the macroscopic observation of wounds and histological evaluation.

## 2. Materials and Methods

### 2.1. Materials

Gelatin (from porcine skin, gel strength 300, Type A), HA (from Streptococcus equi, Mw: 130,000–150,000 g/mol), and CMCS (deacetylation degree: 90%) were purchased from Sigma-Aldrich, Inc. (St. Louis, MO, USA) and Santa Cruz Biotechnology, Inc. (Dallas, TX, USA), respectively. 4-(4,6-Dimethoxy-1,3,5-triazin-2-yl)-4methylmorpholinium chloride (DMT-MM, FUJIFILM Wako Pure Chemical Corp., Tokyo, Japan) was used for the condensation reaction among gelatin, HA, and CMCS. NI was obtained from the Korean Cell Line Bank (Seoul, Korea). Dulbecco’s Modified Eagle Medium (DMEM), bovine serum, penicillin, and streptomycin were purchased from Thermo Fisher Scientific (Waltham, MA, USA). CCK-8 was supplied by Dojindo Molecular Technologies, Inc. (Rockville, MD, USA). Human KGF/FGF-7 Quantikine^®^ ELISA Kit was purchased from R&D Systems (Minneapolis, MN, USA). All organic chemicals were used for further purification.

### 2.2. FDs Comprising Gelatin, CMCS, and HA (GHC-FDs)

#### 2.2.1. Preparation

The FDs were prepared by blending various concentrations of gelatin, HA, and CMCS (Table 1). Briefly, gelatin was dissolved in distilled water at 50 °C. CMCS and HA were dissolved in distilled water at room temperature. Then, the three polymer solutions were mixed, poured into custom-made Teflon molds (15 cm × 15 cm × 7 cm), and maintained at room temperature until pudding. After lyophilization, the spongy FDs were reacted for 1 d using DMT-MM (2 g) as a condensation agent. Subsequently, the FDs were washed several times with distilled water to remove DMT-MM and lyophilized. The freeze-dried FDs were cut into 1 cm × 1 cm pieces and sterilized using ethylene oxide gas. The surface morphologies were observed by using field emission SEM (FE-SEM; Inspect F; FEI, Hillsboro, OR, USA) at an accelerating voltage of 20 kV. Prior to this observations, all FDs were placed on aluminum stubs and coated with Pt by using an ion sputter-coater (Eiko IB-3, Eiko Engineering Co. Ltd., Ibaraki, Japan). The FDs were observed at 200×.

#### 2.2.2. NIH/3T3 Cell Proliferation and Live and Dead Assays

NIH/3T3 cells (5 × 10^6^/each well) were seeded on the surface of hydrogelated FDs in Table 1 and cultured with a medium made of DMEM, 10% bovine serum, 100 U/L penicillin, and 100 mg/mL streptomycin. The cells were incubated for 1, 3, 5, and 7 d at 37 °C under 5% CO_2_. Ten milligrams of CCK-8 were added to each well plate and incubated for an additional 4 h. The supernatant was measured at 450 nm wavelength. Live and dead assay for observing alive and dead cells were investigated by confocal microscope (LSM800 w/Airyscan, Carl Zeiss, Oberkochen, Germany) for 1, 3, and 7 days of culture. At the determined time intervals, cell-cultured FDs were rinsed with PBS three times. A total of 2 μM of Fluorescent marker Calcein AM and 4 μM of ethidium homodimer (EthD-1) were used for staining alive and dead cells, respectively. Alive and dead cells were observed in a green color using an argon laser (488 nm) and in a red color using a HeNe laser (543 nm). Bright field and fluorescence microscopic images were recorded by a CCD color digital camera (QImaging Retiga 4000R).

### 2.3. Characterization of 2-GHC-FD-1, 2-GHC-FD-4 and 2-GHC-FD-8

#### 2.3.1. Tensile Strength Measurement

Tensile tests were performed at room temperature by using a universal testing machine (UTM 4476, Instron, Norwood, MA, USA) according to ASTM D3574. A specific size of 2-GHC-FD-1 (width: 1 cm and length 3 cm), 2-GHC-FD-4 (width: 1 cm and length 3.3 cm) and 2-GHC-FD-8 (width: 1 cm and length 3.2 cm) were prepared. The test conditions were a speed of 10 mm/min and a chart speed of 20 mm/min, using 5-kgf load cells.

#### 2.3.2. WVTR Test

The WVTR test was performed according to EN 13726-2 “Test methods for primary wound dressing. Part 2: Moisture vapor transmission rate of permeable film dressings After fixing the bottom of the moisture-permeable cup with a flat plate, 20 mL of distilled water was added. The initial weights (*w*_1_) of 2-GHC-FD-1, 2-GHC-FD-4, and 2-GHC-FD-8 were measured, fixed on a cup, and the cups were placed in a thermo-hygrostat. FDs were maintained at 37 °C and below 20% humidity. After 24 h, the weights (*w*_2_) of FDs were measured, and the *WVTR* was determined using the following formula:

$$WVTR=\frac{w_{1}-w_{2}}{m^{2}\cdot 24\;\mathrm{hr}}\times 10^{3}\;g$$

where *w*_1_, *w*_2_, and *m*^2^ indicate the initial weight of FDs, the weight of FDs after the test, and the area of the FDs, respectively.

#### 2.3.3. Absorption Rate Test

The absorption rate test was performed according to EN 13726-1 “Test methods for primary wound dressings. Part 1: Aspects of absorbency. Three types of FDs, 2-GHC-FD-1, 2-GHC-FD-4, and 2-GHC-FD-8 were cut to a size of 2 cm × 2 cm and weighed (*w*_1_). After placing the regular size of FDs in Petri dishes, distilled water of 40-fold greater weight than the initial weight of FDs, heated at 37 °C was added and heated for an additional 30 min. The soaked FDs were weighed after eliminating water on the surface of the FDs, and the absorption rate was calculated using the following formula:

$$Absorption\;rate=\frac{w_{1}-w_{2}}{m^{2}}\;\left(g/{\mathrm{cm}}^{2}\right)$$

where *w_1_*, *w*_2_, and *m*^2^ indicate the initial weight of FDs, the weight of FDs after the test, and the initial area of the FDs, respectively. Retention capacity was calculated according to following formula:

$$Retention\;capacity=\frac{D-A}{A}\;\left(g/{\mathrm{cm}}^{2}\right)$$

where D, A indicate initial weight and retention weight of FD, respectively.

#### 2.3.4. Animal Intradermal Reactivity Test

For intradermal reactivity tests of FDs (2-GHC-FD-1, 2-GHC-FD-4 and 2-GHC-FD-8), two types of solvents, sterilized physiological saline as a polar solvent and vegetable soybean oil as a nonpolar solvent, were used for the elution of the FDs, which was evaluated using Table 2. In the case of the soybean oil, it was filtered using a 0.2 μm syringe filter for sterilization. The two solvents were used as the controls. First, a specific weight of FDs (0.5 g/each FD, solvent: 10 mL) was sterilized using ethylene oxide followed by elution at 37 °C for 72 h. The hair on the back of the experimental rats (*n* = 6 for each FD) were removed using a razor and depilatory cream and cleaned with an alcohol swab. A specific volume (1 mL) of the control and experimental elution were intradermally injected. Polar solvent-derived and nonpolar-derived eluents were intradermally injected into the left and right back parts, respectively. After intradermal injection, the eluent-treated parts were carefully monitored for erythema, crusting, and edema.

### 2.4. Preparation of FDs Containing Various Concentrations of FGF-7

As presented in Table 3, various concentrations of FGF-7 solutions were soaked in sterilized FDs (1 cm × 1 cm) made of 2% (*w*/*v*) gelatin, 10% (*w*/*w*) HA (concentration calculated from the weight of gelatin), and 50% (*w*/*w*) CMCS (concentration calculated from the weight of gelatin), which was designated as 2-GHC-FD-8/F1~6. After lyophilization, FDs were used without further modification. All FDs were observed using an SEM (Bio-LV SEM, S-2250N, Hitachi, Japan). Freeze-dried FDs were placed on the mount using carbon tape. Before SEM observation, all FDs were coated with Ag/Pd by using a plasma sputter (Emscope SC500K, London, UK). The FDs were observed at 200×.

### 2.5. Fourier Transform Infrared Spectroscopy (FTIR)

The FDs, shown in Table 3, were recorded on a Spectrum GX FT-IR spectrometer (PerkinElmer, Boston, MA, USA). FTIR spectra was monitored from 400 to 4000 cm^−1^ with 256 scans and a resolution of 2 cm^−1^.

### 2.6. Release Test of FGF-7

FGF-7-loaded 2-GHC-FD-8/F1-6 were placed in Petri dishes (90 mm × 15 mm), and PBS (pH 7.4) was added. The FDs were incubated at 37 °C to determine the release periods. At each time interval (10, 20, 30 min, and 1, 2, 4, 8, 12, 24, 36, 48 h), 1 mL of eluate from each sample was extracted, and the same volume of fresh PBS was added. All eluates were evaluated using the Human KGF/FGF-7 Quantikine^®^ ELISA Kit (R&D Systems, Minneapolis, MN, USA) according to the manufacturer’s protocol. Briefly, all eluates were diluted 10,000-fold by using the RD5R buffer of the kit. The diluted eluates were transferred to a 96-well plate and incubated at room temperature for 3 h. The eluates were measured at 450 nm by using a microplate reader, according to the manufacturer’s protocol.

### 2.7. In Vivo Animal Study

Forty-eight Balb/C mice (20 ± 3.2 g, 6 weeks, mice per each group = 6; G-bio, Gwangju, Republic of Korea) were used to investigate skin wound healing [21,22]. Prior to this experiment, mice carried in animal laboratory was stabilized for 7 days in a breeding facility set at 22 ± 3 °C and a humidity ranging from 30 to 70%. After anesthetizing using ketamine hydrochloride (Ketara^®^; 50 mg/kg, YUHAN, Seoul, Republic of Korea) and Xyalizine hydrochloride (Rompun^®^; 5 mg/kg, Bayer Healthcare, Seoul, Republic of Korea), the back of each mouse was shaved, and the specific size of the wound (diameter: 1 cm) was produced using a biopsy punch. The wounds were entirely covered with 2-GHC-FD-8 and 2-GHC-FD-8/F1-6, and protected with Tegaderm^TM^ (St. Paul, MN, USA) and Coban^TM^ (St. Paul, MN, USA) to minimize infection. During the experiment, mice was randomly divided in eight groups, including sham (control): group I (sham), group II (2-GHC-FD-8), group III (2-GHC-FD-8/F1), group IV (2-GHC-FD-8/F2), group V (2-GHC-FD-8/F3), group VI (2-GHC-FD-8/F4), group VII (2-GHC-FD-8/F5), and group VIII (2-GHC-FD-8/F6). At each time interval (days 7, 14, and 21), the wound size of each mouse was measured using the ImageJ program (National Institutes of Health, Bethesda, MD, USA). After 21 days, mice were sacrificed for histological evaluations.

### 2.8. Hematoxylin and Eosin (H&E) and Masson’s Trichrome (MT) Staining

At twenty-one days after surgery, the mice used for the test were euthanized using 100% CO_2_, and their skin tissues, including 2-GHC-FD-8 and 2-GHC-FD-8/F1-6-treated wounds, were removed. The tissues were fixed in 10% neutral-buffered formalin for 24 h. For removing residual formalin, the tissue samples were washed with 0.1 M PBS (pH 7.4) for 20 min. Dehydration was conducted in a series of ethanol solutions from 70 to 100%. The dehydrated tissues were embedded in paraffin and sectioned at 4 µm. For evaluating histopathological changes and collagen formation, the slides were stained with H&E (ab ab245880, Abcam, Cambridge, UK) and MT (TRM-2, ScyTek, West Logan, UT, USA) according to manufacturer’s guidelines, respectively [23]. There were twenty-four wounds per mouse per condition because twelve mice per each group with four wounds on its back per each mouse were used. Each groups included a total of three stained slides for H%E and MT, and the histological evaluations were blindly performed. Three fields per each slide were observed.

The stained slides were visualized using a fluorescence microscope (AX70, TR-62A02, Olympus, Tokyo, Japan) at 2.0× (500 μm). The results were read by three histopathologists.

### 2.9. Statistical Analysis

All quantitative data were statistically analyzed using one-way analysis of variance (ANOVA) of Origin program (OriginLab^®^, Northampton, MA, USA) followed by a post-hoc test because all data were normally distributed. The normality test of distribution was assessed using the Shapiro-Wilk test provided by the SPSS software ISPSS Inc., Chicago, IL, USA). Quantitative data were analyzed five times and expressed as the mean ± standard deviation (* *p* < 0.05). The number of each sample for the animal test was determined using MedCalc Statistical Software (MedCalc Software bvba, Ostend, Belgium), using α (*p* = 0.05) and power (1β = 0.8).

## 3. Results and Discussion

### 3.1. Preparation and Characterization of Gelatin, CMCS, and HA-blended Foam Dressings (GHC-FDs)

#### 3.1.1. Preparation of FDs

Table 1 shows the blending ratios of gelatin, HA, and CMCS for the preparation of FDs. This blend was chemically crosslinked among natural hydrogels through an amide bond [15,16]. The carboxyl and amine groups of gelatin, the carboxyl group of HA, and the amine group of CMCS are related to amide bond formation through condensation reactions among the functional groups [24,25]. Hydrogelation was not observed in the nine samples prepared from 1% (*w*/*v*) gelatin; it was observed in 27 samples containing 2, 5, and 10% (*w*/*v*) gelatin. In our study, gelatin concentration significantly influenced hydrogelation. Therefore, the absence of hydrogelation at 1% (*w*/*v*) gelatin may be attributable to the relatively low concentration of gelatin related to amide bond formation. The FD samples designated as 2-GHC-FD-1-9, 5-GHC-FD-1-9, and 10-GHC-FD-1-9 for further experiments.

#### 3.1.2. Observation of Surface Morphology

**Figure 2:**
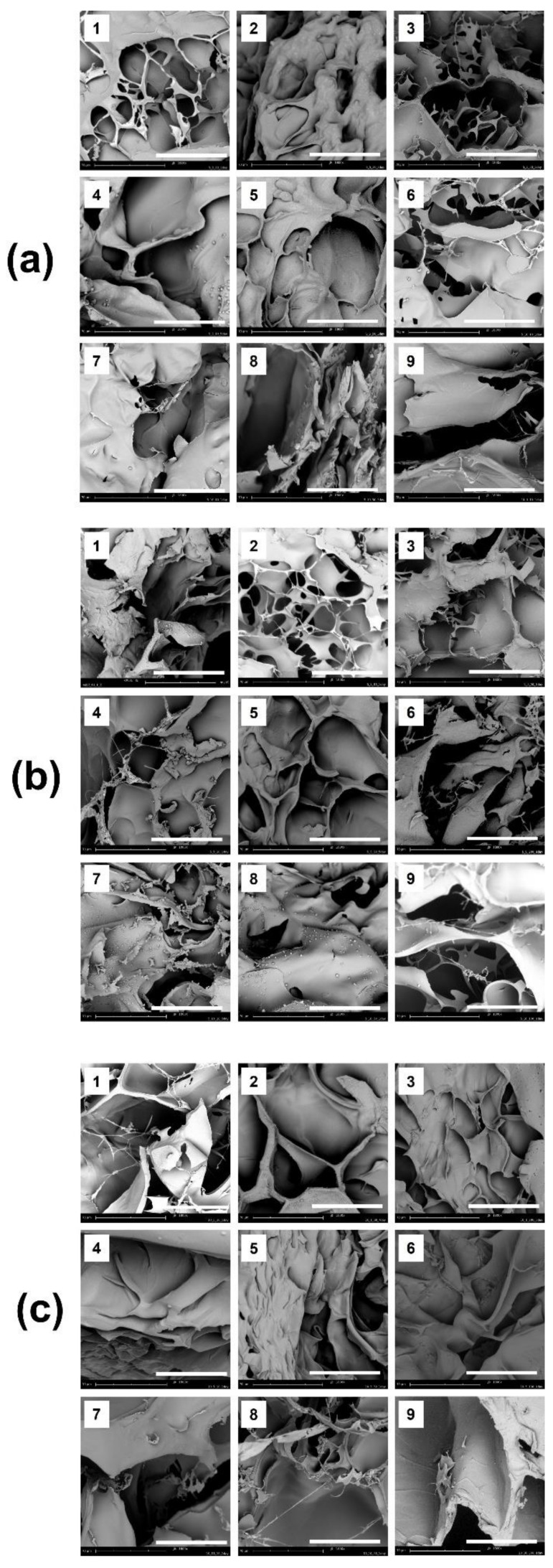
Surface morphologies of lyophilized 2-GHC-FD-1-9, 5-GHC-FD-1-9, and 10-GHC-FD-1-9 observed by FE-SEM at 1800×. (**a**) 2-GHC-FD-1-9, (**b**) 5-GHC-FD-1-9, and (**c**) 10-GHC-FD-1-9. White scale bars indicate 70 μm.

After lyophilization, the surface morphologies of the freeze-dried FDs, were observed using SEM (Figure 2). The porous structure of biomaterials for wound healing is a vital factor because it offers cell infiltration, high permeability, and diffusion of oxygen and nutrients [26]. Porous structures were observed in all samples, regardless of the concentrations of gelatin, HA, and CMCS, due to the lyophilization of the occupied water molecules.

#### 3.1.3. NIH/3T3 Cell Proliferation

**Figure 3:**
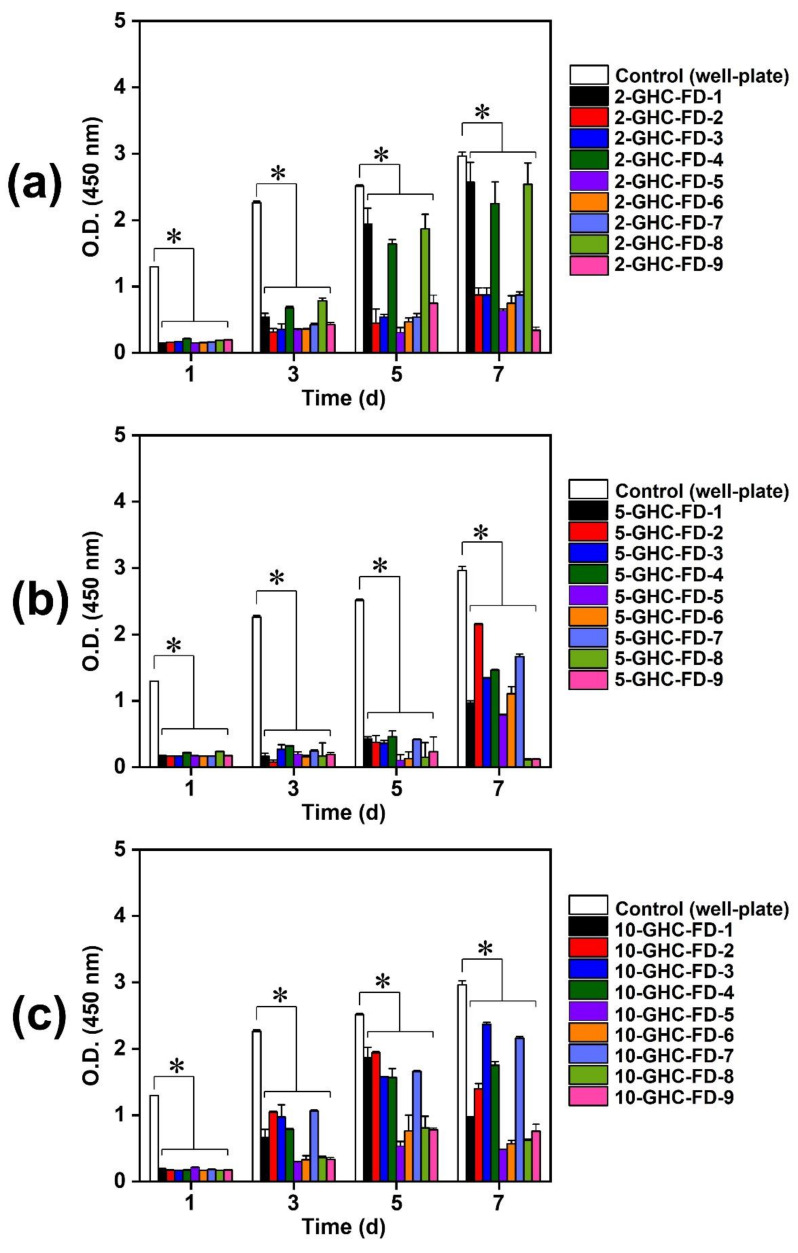
O.D. values of (**a**) 2-GHC-FD-1-9, (**b**) 5-GHC-FD-1-9, and (**c**) 10-GHC-FD-1-9 measured by CCK-8 assay at 450 nm. The test was performed for 1, 3, 5, and 7 days (*n* = 5). As a control, O.D. of cells cultured on well-plate was used. The statistical significance was indicated by an asterisk (*) for *p* < 0.05. The FD samples were compared to control (well-plate). At (**a**–**c**) graphs, the FD samples had significant differences as compared to control (well plate). The type of this result is involved in continuous data.

The cell proliferation results analyzed by the CCK-8 assay are shown in Figure 3. Gelatin is produced by heating collagen composed of Arg-Gly-Asp (RGD), resulting in the formation of a random-coiled domain. Therefore, it allows for the adhesion, spreading, and proliferation of cells [27,28]. CMCSs have been extensively investigated as wound dressings as they can improve cell adhesion and proliferation, and possess excellent biological functions, including antibacterial and biomimetic properties [10,29]. HA is a naturally occurring glycosaminoglycan (GAG) and a main component of the extracellular matrix (ECM). These characteristics quickly induce cell adhesion [30]. Compared with control, GHC-based samples remarkably exhibited significant difference in the cell proliferation. The FDs related to 2%, 5%, and 10% (*w*/*v*) gelatin (2-GHC-FD-1–9, 5-GHC-FD-1–9, and 10-GHC-FD-1–9) had relatively lower cell proliferation than those in the control (well plate). This finding may be attributed to the difference in cellular behavior between two- and three-dimensional networks. Throughout the culture periods, compared with control (well-plate), the FDs had a significant difference in cell proliferation. The proliferation of all FDs gradually increased over 5 d, but cell proliferation after 5 d was complicated. In 2-GHC-FD-1–9, FDs gradually improved cell proliferation throughout the culture period. However, in 5-GHC-FD-1–9 and 10-GHC-FD-1–9, FDs (5-GHC-FD-5, 6, 8, 9 and 10-GHC-FD-5, 6, 8, 9) with a relatively large amount of HA and CMCS exhibited decreased cell proliferation. These results can be ascribed to the effects of the mechanical properties of FDs.

### 3.2. Characterizations of 2-GHC-FD-1, 2-GHC-FD-4, and 2-GHC-FD-8

**Figure 4:**
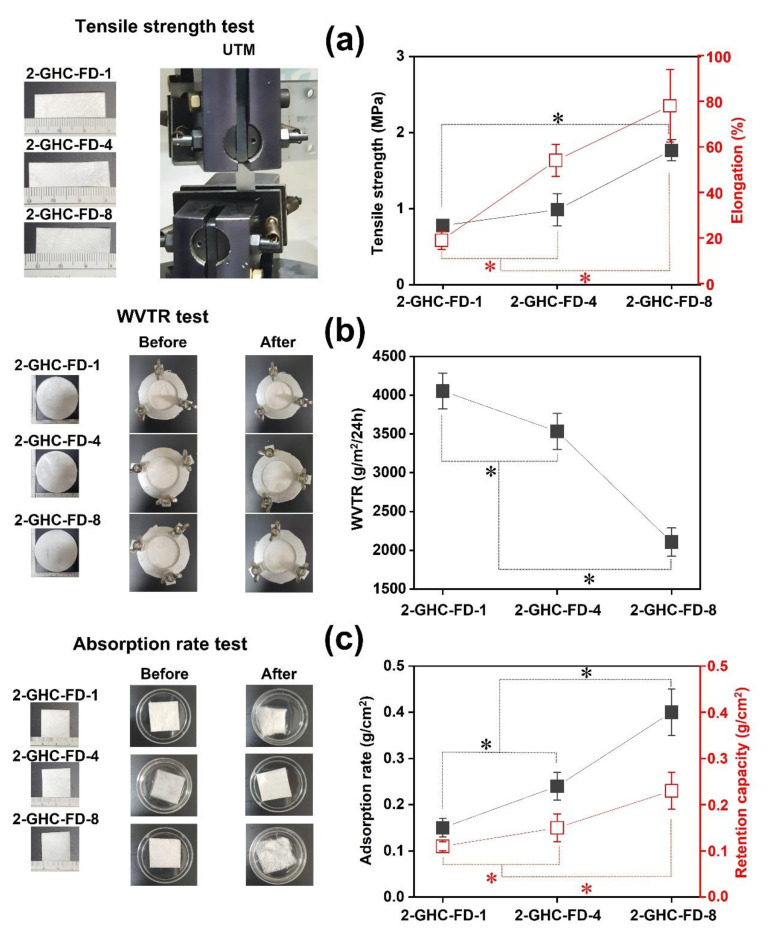
(**a**) Tensile strengths and elongations, (**b**)WVTR, and (**c**) absorption rate and retention capacity of 2-GHC-FD-1, GHC-FD-4, and GHC-FD-8. The test was performed five times. The statistical significance was indicated by an asterisk (*) for *p* < 0.05. The results of FD samples compared each other. In the tensile strength of (**a**) graph, 2-GHC-FD-8 had significant differences as compared to 2-GHC-FD-1. The elongation result of 2-GHC-FD-8 had significant differences as compared to those of 2-GHC-FD-1 and 2-GHC-FD-4. In WVTR results of (**b**) graph, 2-GHC-FD-8 had significant differences as compared to 2-GHC-FD-1 and GHC-FD-4. In (**c**) graph, the adsorption rate and retention capacity of 20GHC-FD-8 had significant differences as compared to those of 2-GHC-FD-1 and 2-GHC-FD-4. The type of this result is involved in continuous data.

The three types of FDs were selected based on the results of the afore-mentioned cell proliferation test. Evaluating mechanical properties, such as tensile strength, is a significant factor in preparing biomaterial scaffolds in various tissue-engineering fields. Ideal biomaterial scaffolds in tissue engineering have mechanical properties similar to those of native tissue or organs intended for regeneration. For example, native skin has a tensile strength between 5 and 30 MPa and an elongation between 35 and 115% [31,32]. This may indicate that cells related to skin generation actively proliferate in biomaterial scaffolds adjusted to the tensile strength and elongation of native tissue. In Figure 4a, various tensile strengths and elongations were confirmed according to the concentration variables of gelatin, HA, and CMCS. Specifically, 2-GHC-FD-1, 2-GHC-FD-4, and 2-GHC-FD-8 exhibited tensile strengths below 5 MPa. Furthermore, 2-GHC-FD-1 and 2-GHC-FD-4 exhibited shorter elongation (19% and 54%) than 2-GHC-FD-8 (78%). The results of 2-GHC-FD-8 had a significant difference as compared to those of 2-GHC-FD-1 and 2-GHC-FD-4. These results may imply that 2-GHC-FD-8 is a more appropriate scaffold than 2-GHC-FD-1 and 2-GHC-FD-4 for wound healing.

An optimal moisture environment is essential for wound healing acceleration without causing substantial side effects [33,34]. This wetted condition leads to the acceleration of re-epithelialization, reduction of inflammation, necrosis, and scar formation [1]. Furthermore, wound fluid in a wetted condition stimulated the proliferation of keratinocytes and fibroblasts for wound repair [1]. Therefore, ideal wound dressings must provide adequate wetting conditions to prevent dehydration. Lee et al. explained that a three-dimensional wound dressing with approximately 2028.3 g/m^2^/24 h provides an optimal moisture environment for improving the proliferation and function of fibroblasts and epidermal cells [35]. The WVTR results of the FD samples are shown in Figure 4b. 2-GHC-FD-1 and 2-GHC-FD-4 had the WVTR of 4054.4 and 3532.2 g/ m^2^/24 h, respectively, on the other hand, 2-GHC-FD-8 exhibited 2105.3 g/m^2^/per 24 h. The results of 2-GHC-FD-8 had a significant difference as compared to those of 2-GHC-FD-1 and 2-GHC-FD-4. This result indicates that 2-GHC-FD-8 could be an optimal scaffold for wound healing acceleration.

The absorption of exudate from the wound influences wound healing. If the absorption rate is low, the exudate remains in the wound, causing skin maceration and bacterial infection [2,35]. Therefore, balancing the absorption rate and retention capacity of the exudate in wound dressings must be appropriately controlled. As exudates are formed differently depending on the wound type and healing stage, wound dressing must be chosen by considering the rate of exudate formation to avoid drying-out or maceration of wounds [3]. The absorption rate/retention capacities of 2-GHC-FD-1, 2-GHC-FD-4, and 2-GHC-FD-8 are shown in Figure 4c. The results of 2-GHC-FD-8 had a significant difference as compared to those of 2-GHC-FD-1 and 2-GHC-FD-4. The absorption/retention capacities of 2-GHC-FD-8 were noticeably higher than those of 2-GHC-FD-1 and 2-GHC-FD-4. Their absorption/retention capacities were 0.15/0.11 g/cm^2^, 0.24/0.15 g/cm^2^, and 0.40/0.23 g/cm^2^, respectively.

**Figure 5:**
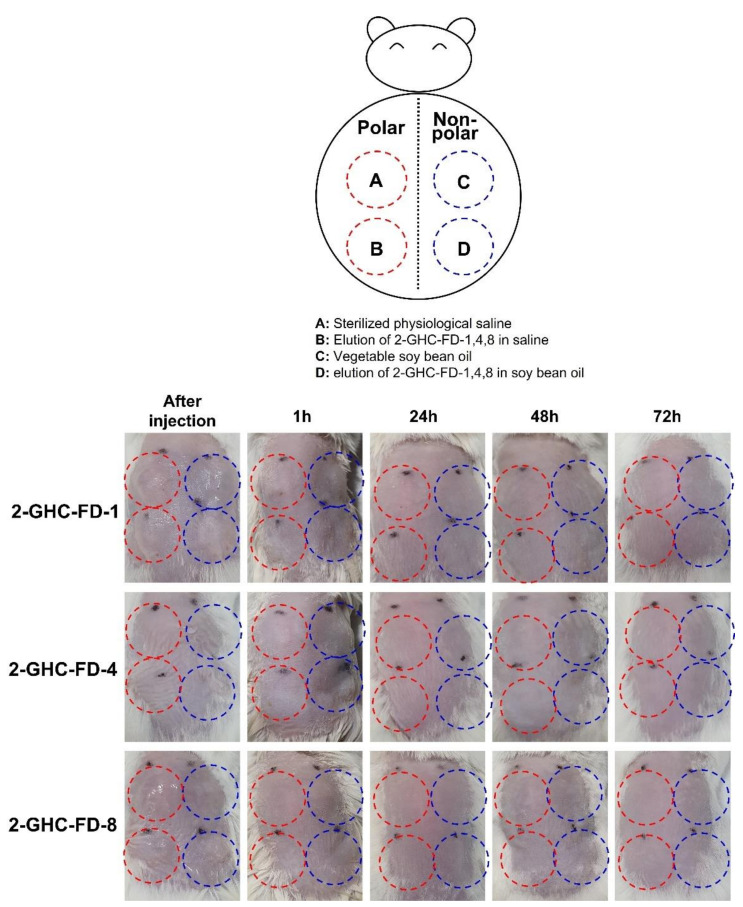
Macroscopic appearances of backs of mice injected with elution from 2-GHC-FD-1, 2-GHC-FD-4, and 2-GHC-FD-8. This test was performed for 1, 24, 48, and 72 h after injection. Red dotted circles–(**A**) sterilized physiological saline; (**B**) elution of 2-GHC-FD-1,4,8 saline. Sky blue dotted circles–(**C**) vegetable soy bean oil; (**D**) elution of 2-GHC-FD-1,4,8 in soy bean oil. The black points indicate injection position.

This test was conducted using ISO-10993-10 ““Biological evaluation of medical devices: Tests for irritation and skin sensitization.” It is a biological stability evaluation of medical devices applied to the skin, eyes, and mucous membranes, and must be performed. Figure 5 and Table 4 shows the results of the intradermal reactivity test using FDs for 1, 24, 48, and 72 h. As shown in Figure 4, no abnormal skin responses were observed in the FDs. For a further investigation of the intradermal responses, the degree of erythema, incrustation, and edema was determined in polar and nonpolar solvents. The findings demonstrated no abnormality in the solvents.

### 3.3. Characterization of FGF-7-Loaded 2-GHC-FD-8 (2-GHC-FD-8/F) 

As a platform for loading FGF-7, 2-GHC-FD-8 was selected based on the afore-mentioned cell proliferation, animal intradermal reactivity, WVTR, and absorption rate tests. The 2-GHC-FD-8 containing FGF-7 ranging from 2 × 10^−11^ M to 64 × 10^−11^ M designated as 2-GHC-FD-8/F1-6, as shown in Table 3.

#### 3.3.1. FTIR Spectra

**Figure 6:**
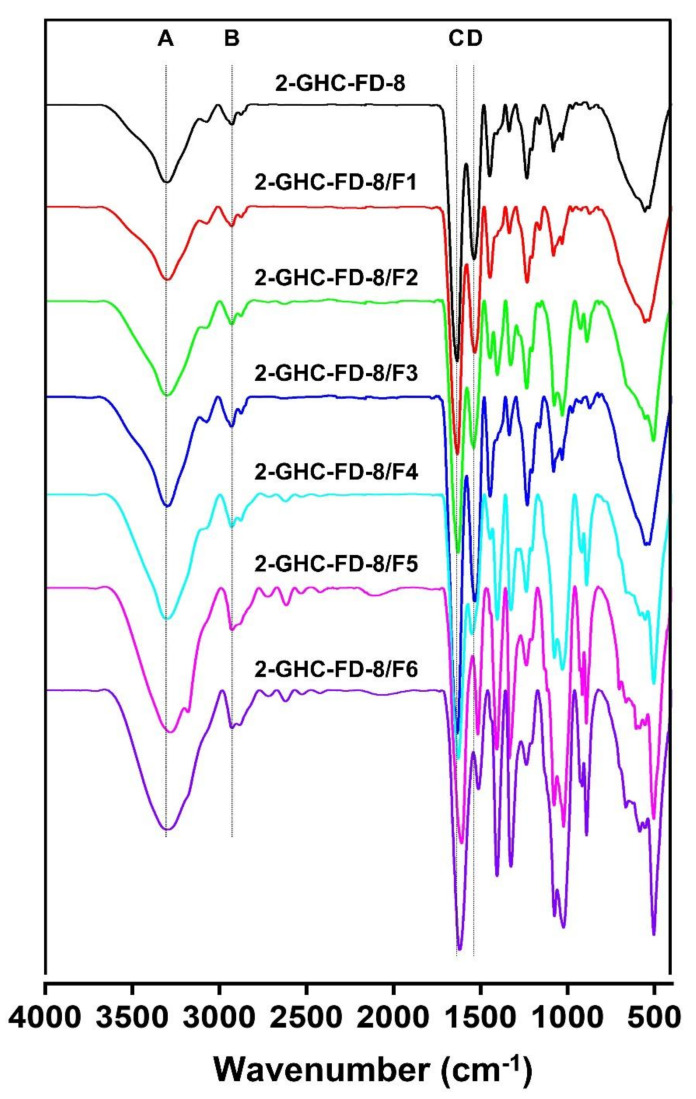
FTIR spectra of 2-GHC-FD-8/F1-6 monitored from 400 to 4000 cm^−1^. A, B, C, and D indicate OH, CH, C=O/amide I, and amide II signals, respectively.

FTIR is a widely used analytical method for analyzing structural properties of materials and a pivotal technique for characterizing polymeric and biopolymeric materials [36]. Gelatin, CMCS, and HA commonly possess functional groups, including OH (3303 cm^−1^), CH (2926 cm^−1^, C=O/amide I (1620 cm^−1^), and amide II (1511 cm^−1^). The three polymers also have functional groups related to amides I and II. Besides the common groups, NH_2_ groups exist in the backbone of gelatin. Loading of various concentrations of FGF-7 in 2-GHC-FD-8 was investigated by a FTIR analysis, as shown in Figure 6. 2-GHC-FD-8/F1~6 showed an increased intensity in the functional groups, as compared to 2-GHC-FD-8. This finding can be attributed to the loading of FGF-7.

#### 3.3.2. Observation of Surface Morphology and Porosity

**Figure 7:**
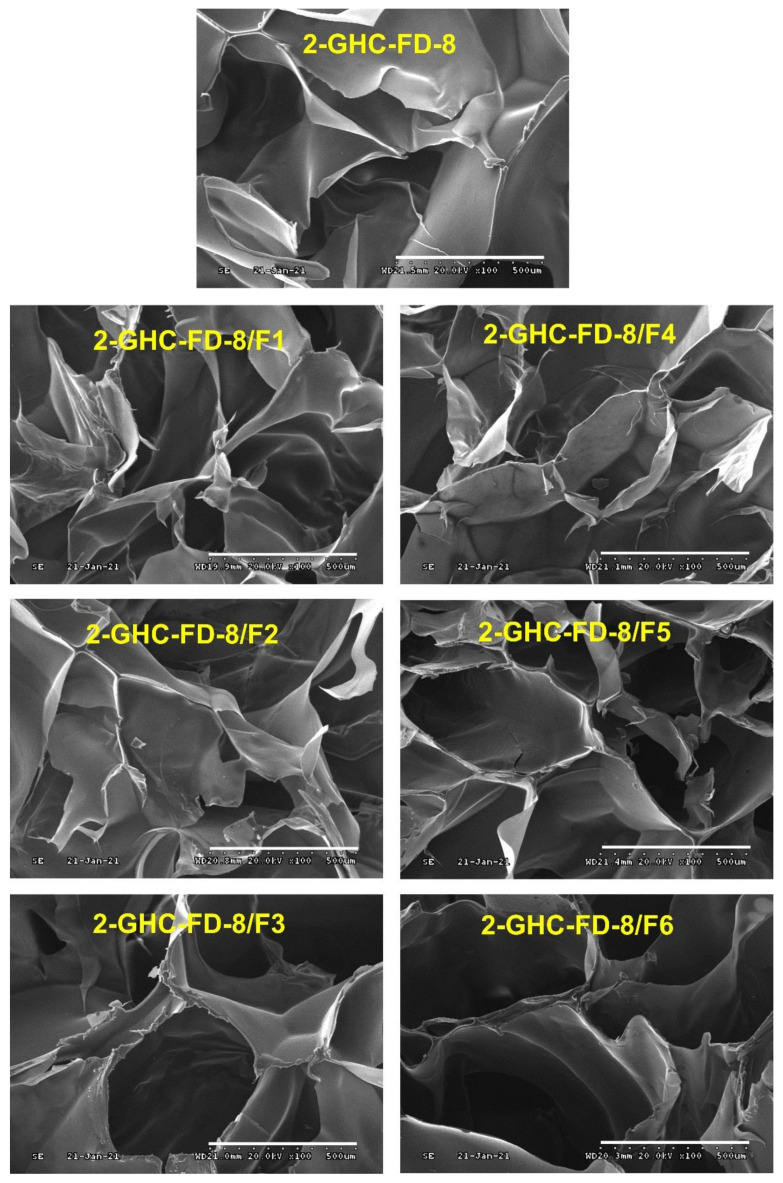
Surface morphologies of 2-GHC-FD-8/F1-6 observed by SEM at 100×. White scale bars indicate 500 μm.

Surface morphologies of the freeze-dried GHC and 2-GHC-FD-8/F1–6 were observed using SEM (Figure 7). All FDs showed interconnected porous structures. This surface characteristic mimics naturally occurring tissue structures, promoting the exchange of gases and nutrients for cell proliferation [37]. To improve cell growth, adhered cells absorb nutrients and remove metabolites via pores. This pore size must be adequately controlled because cells cannot migrate into scaffolds with tiny pores and adhere to scaffolds with large pores [37]. All FDs showed similar pore sizes, indicating that cell proliferation was not affected by the pore size.

#### 3.3.3. Live and Dead Assay

**Figure 8:**
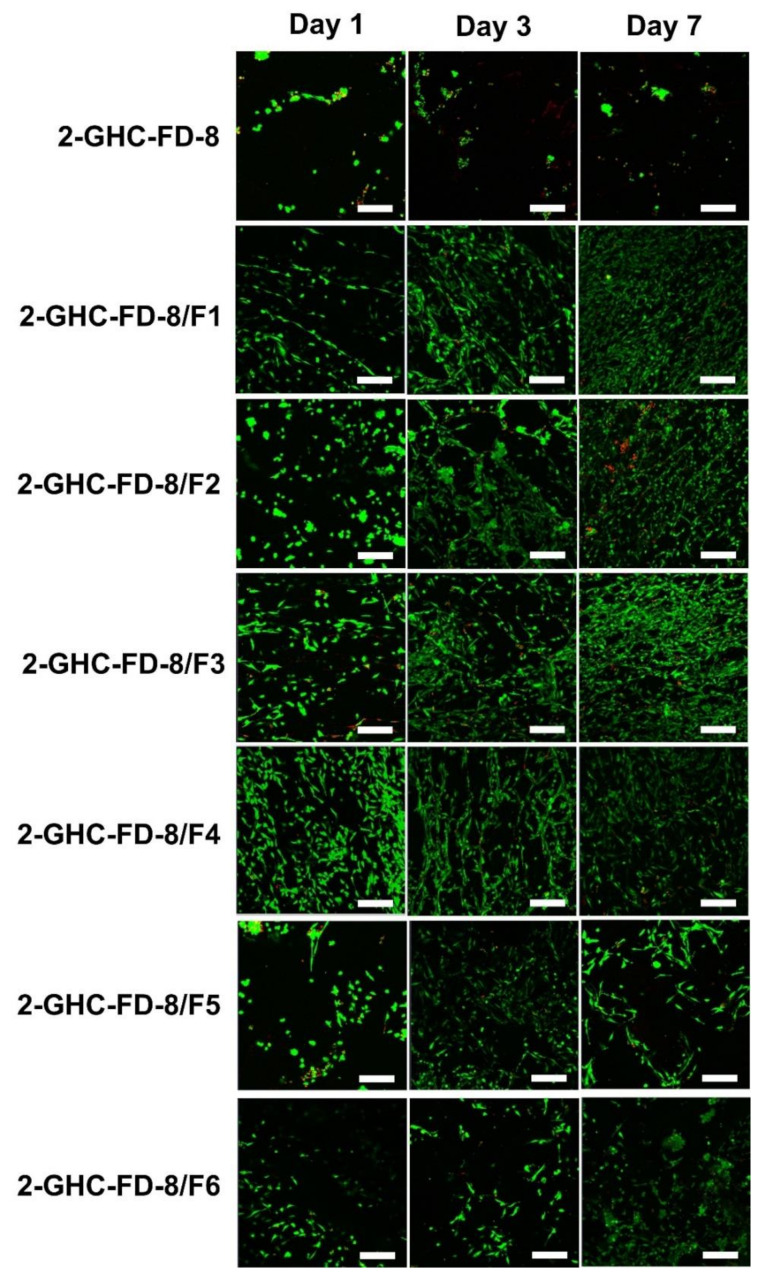
Confocal images of NIH/3T3 cells cultured on 2-GHC-FD-8 and 2-GHC-FD-8/F1-6 for 1, 3, and 7. The green and red colored cells indicate live and dead cells, respectively. White bars indicate 60 μm. Magnification is 10×.

Figure 8 shows the cell proliferation of NIH3T3-E1 cells cultured on 2-GHC-FD-8 and 2-GHC-FD-8/F1~6, investigated by live/dead assay. Cell proliferation on 2-GHC-FD-8 was used as a control. Throughout the culture period, alive cells were markedly present in 2-GHC-FD-8/F1-3. Above all, cells seemed to be actively proliferated in 2-GHC-FD-8/F3. Frederic et al. found that cells proliferate gradually until a specific FGF-7 concentration of 7.5 × 10^−11^ M, and the proliferation sharply decreased thereafter [38]. Our results also showed a parabolic cell proliferation, in which cell proliferation increased until 8.0 × 10^−11^ M and decreased thereafter. In addition, 2-GHC-FD-8/F1~6 exhibited higher cell proliferation rate than 2-GHC-FD-8. The results showed that cell proliferation was affected by the FGF-7 concentration. In addition, it was proven that an adequate concentration of FGF-7 is a prerequisite for improving fibroblast proliferation, despite FGF-7 being related to fibroblast proliferation.

### 3.4. Release Behavior of FGF-7

**Figure 9:**
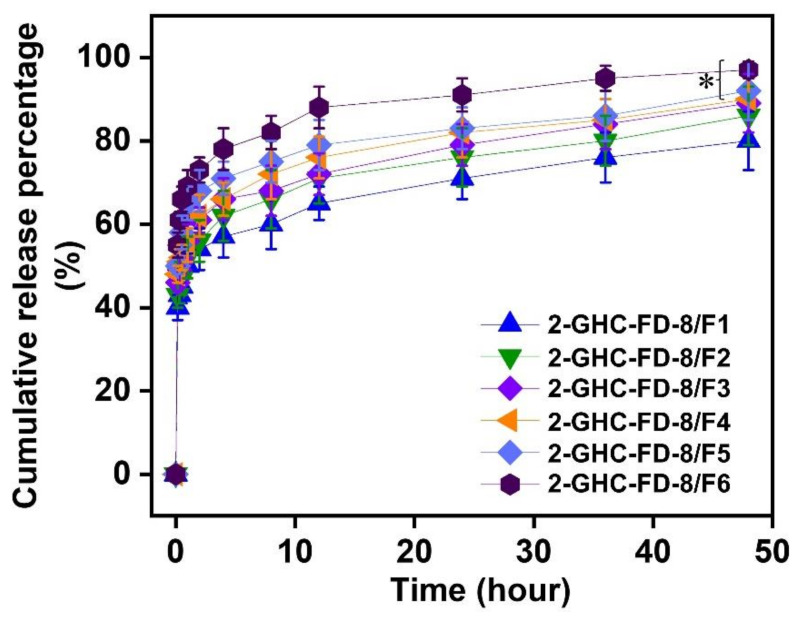
Cumulative release percentage of FGF-7 in 2-GHC-FD-8/F1-6 measured at 37 °C for 48 h. The test was performed five times. The statistical significance was indicated by an asterisk (*) for *p* < 0.05. At 48 h, 2-GHC-FD-8/F5-6 has significant differences as compared to 2-GHC-FD-8/F1. This type of result is involved in continuous data.

A controlled release of growth factors in a sustained manner can improve the proliferation and differentiation of cells, consequently accelerating wound healing [39]. Scaffolds of the porous structure provide a controlled release of growth factors, followed by applications of tissue engineering [40,41,42]. In Figure 9, porous 2-GHC-FD-8, in common with the afore-mentioned reports, displayed two release patterns: Initial burst and sustained release. In 2-GHC-FD-8/F1~6, FGF-7 was rapidly released within 1 h and released sustainably for 48 h. The initial burst and controlled release were caused by FGF-7 on the surface and in the 2-GHC-FD-8 matrix.

### 3.5. Macroscopic Observation and Size Calculation of Remaining Wounds

**Figure 10:**
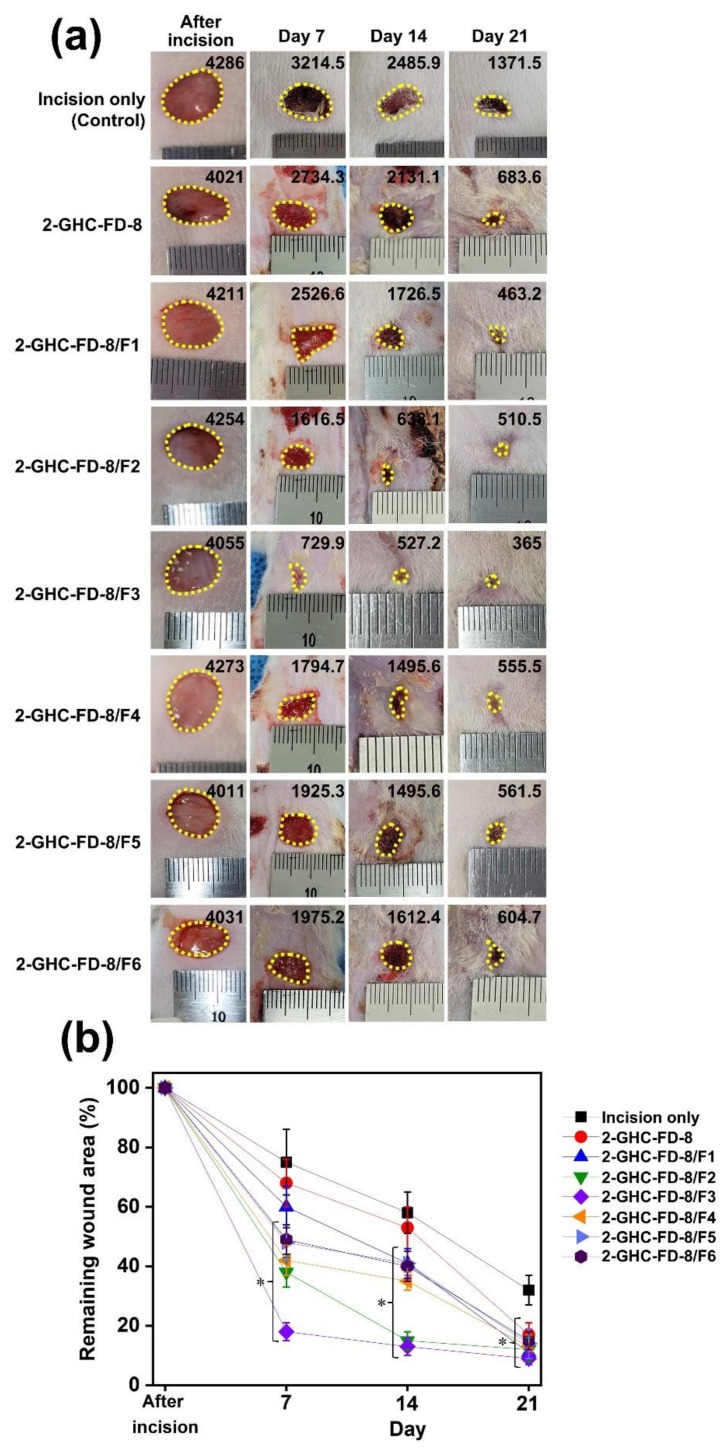
(**a**) Macroscopic appearances; and (**b**) remaining area of wounds treated with 2-GHC-FD-8 and 2-GHC-FD-8/F1-6 for 7, 14, and 21 days. In each photo, the black-colored number of upper-right indicates were calculated by an Image J program, indicating the area of remaining wounds. In (**b**) graph, the statistical significance was indicated by an asterisk (*) for *p* < 0.05. The results of the FD samples were compared to that of incision only. The type of this result is involved in continuous data.

An in vivo animal test for wound healing evaluation was conducted using skin excisional mice as animals can provide abundant, controllable, and accurate information for wound healing studies. In addition, 1 cm of wound size for our animal test was determined because the size of 0.5 cm is automatically recovered within 15 d [43,44]. The wounds treated with 2-GHC-FD-8 and 2-GHC-FD-8/F1–6 were macroscopically observed for 7, 14, and 21 d after surgery, and the remaining wound area was investigated (Figure 10). Although the wounds were produced using biopsy punches with a diameter of 1 cm, practical wound sizes showed a small difference because they were produced manually. The macroscopic appearances of wounds treated with 2-GHC-FD-8 and 2-GHC-FD-8/F1~6 was shown in Figure 10a. The 2-GHC-FD-8 treatment accelerated wound recovery more rapidly than the control. Compared to 2-GHC-FD-8, the addition of FGF-7 to 2-GHC-FD-8 accelerated wound closure, in which the defect size remarkably decreased until FGF-7 increased to 8.0 × 10^−11^ M and was maintained thereafter. These findings indicate that 2-GHC-FD-8 and FGF-7 are important factors for accelerating wound healing. Figure 10b shows the remaining wound area calculated by the Image J program on days 0, 7, 14, and 21 after wound production. Approximately 80% wound closure was observed in the control group after 21 d, and 90% was observed in the 2-GHC-FD-8 group. Notably, 2-GHC-FD-8/F1–3 resulted in more remarkable wound closure than 2-GHC-FD-8/F4–6 for 21 d. In special, 2-GHC-FD-8/F3 decreased wound closure markedly, indicating that 8.0 × 10^−11^ M of FGF-7 is a threshold concentration for wound healing acceleration. The remaining wound area of control, 2-GHC-FD-8, and 2-GHC-FD-8/F1~6 decreased from 100% to 32 ± 5%, to 17 ± 4%, 11 ± 3%, 12 ± 4%, 9 ± 2%, 13 ± 4%, 14 ± 5%, and 15 ± 3%, respectively.

### 3.6. Histological Evaluation

**Figure 11:**
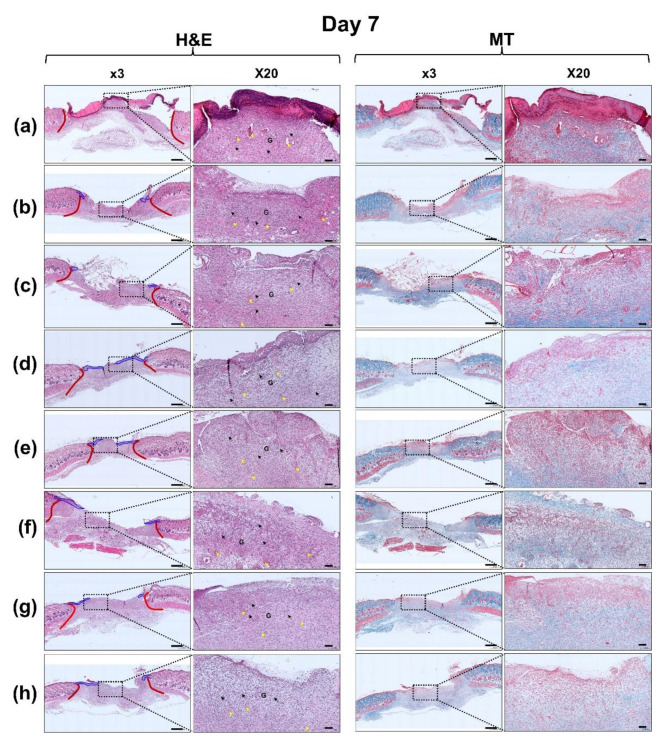

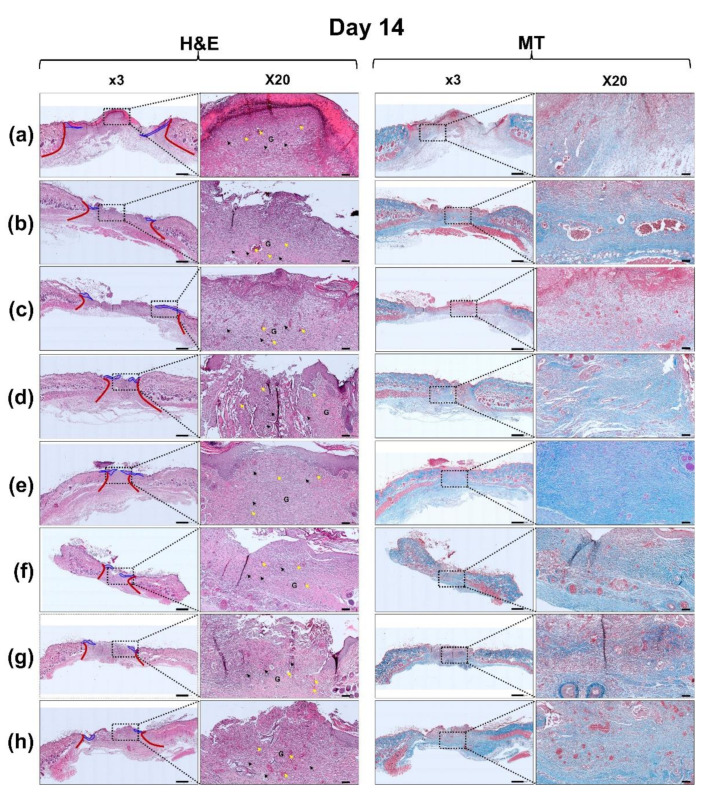

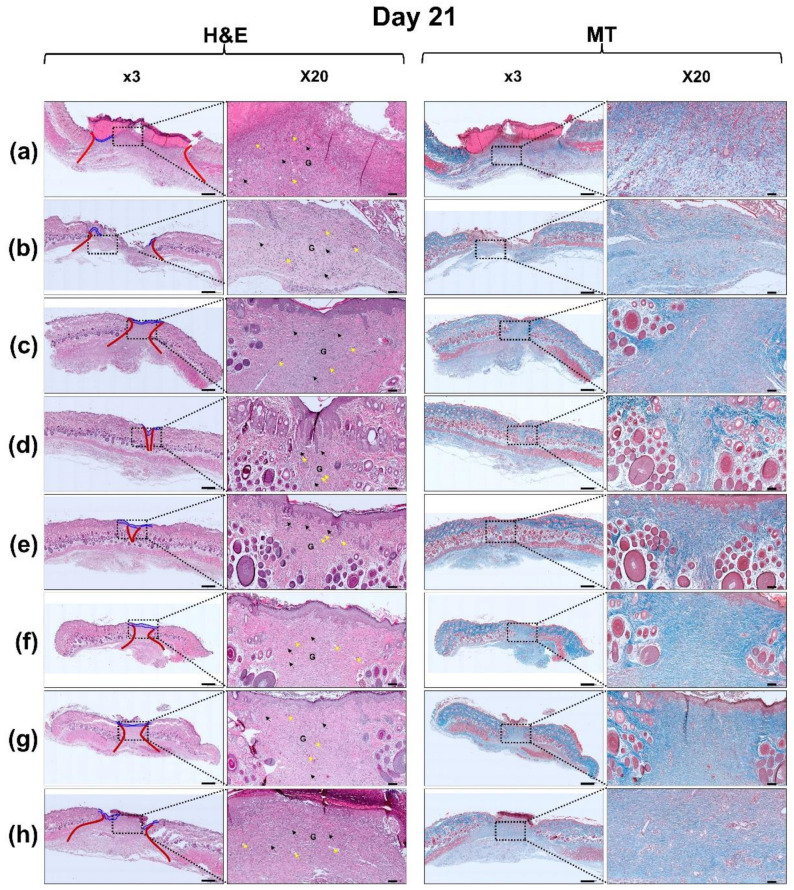
H&E and MT stained slides of wounds treated with (**a**) 2-GHC-FD-8; (**b**) 2-GHC-FD-8/F1; (**c**) 2-GHC-FD-8/F2, (**d**) 2-GHC-FD-8/F3; (**e**) 2-GHC-FD-8/F4; (**f**) 2-GHC-FD-8/F5; (**g**) 2-GHC-FD-8/F6; and (**h**) 2-GHC-FD-8/F7 for 7, 14, and 21 days. At slides at ×3, red and blue lines indicate boundary between normal and newly formed tissues, and newly formed epithelial layer, respectively. At slides at ×20, black and yellow arrows indicate inflammatory cells and newly formed blood vessels, respectively. G: granulation tissue.

Histopathological changes in the control and all hydrogel-treated wounds for 7, 14, and 21 d were investigated using H&E and MT staining (Figure 11). Angiogenesis is an important factor for wound healing [45]. This new angiogenesis is usually involved in the proliferative phase of wound healing process, which is produced by the action of endothelial cells and various cytokines such as FGF and vascular endothelial growth factor (VEGF) [46]. 

On day 7, 2-GHC-FD-8-based samples showed more noticeable angiogenesis than control. In addition, neo-epidermis was not observed in control, while the FD samples induced neo-epidermis from the wound edge. Due to the absorption capacity of fluids of gelatin, it can prevent fluid accumulation from wounds. By absorbing excess exudate and cell debris from wounds, gelatin may improve wound healing [47]. HA affects the signaling of epidermal and dermal cells [48]. CD44, as HA receptor present on the surface of cell membranes, triggers differentiation in human keratinocytes and fibroblast cells [49,50,51]. Therefore, HA attached to wounds may bind to CD44 in epidermal or fibroblast cells and wound healing may be promoted by the signal transduction of CD44. In addition, it is known that CMCS accelerates granulation tissue formation by promoting the proliferation of fibroblasts and keratinocytes followed by wound healing acceleration [52]. Fibroblasts lead to the reorganization of extracellular matrix by the synthesis of structural proteins and secretion of cytokines [53]. Subsequently, CMCS diminishes scar formation by regulating collagen deposition from stimulating IL-8 in fibroblasts, TNF-a in macrophages and MMP-2 in wound skin. 

On days 7 and 14, all slides exhibited wound healing phases, including the inflammatory and proliferative phases. A few inflammatory cells and granulation tissue formation were observed in all H&E-stained images at a magnification of × 50 (Day 7 and 14 of Figure 11. We found that FGF-7 noticeably accelerated re-epithelization. Furthermore, re-epithelization was distinctly observed in 2-GHC-FD-8/F1~6. After 21 d, in all samples, inflammatory cells were decreased (Day 21 of Figure 11). In addition, bulbs, hair follicles, papillae, and sebaceous glands were confirmed in 2-GHC-FD-8/F2-3-treated wounds. In particular, among all samples, 2-GHC-FD-8/F3 showed the highest number of newly formed blood vessels, indicating its superior wound healing. These results indicate that FGF-7 concentrations less than 8.0 × 10^−11^ M can improve wound healing. Moreover, compared with the control, 2-GHC-FD-8/F3 resulted in a remarkable collagen deposition. Collagen formation in wounds is a very important point for wound remodeling and tensile strength of healed tissues [54]. MT stain was employed for investigating collagen formation in wounds after 7, 14, and 21 days of healing period (Figure 11). The collagen formation was observed in blue region in all samples (Figure 11). Compared with control, 2-GHC-FD-8-based samples displayed marked organed and numerous collagen formation. Furthermore, densely packed and parallel aligned mature collagen formation was observed in 2-GHC-FD-8-based samples.

During wound healing, macrophages play important roles in cleaning wounds and remodeling, including classical activation (M1 macrophages) and alternative activation (M2 macrophages). In the inflammatory phase (M1 macrophages) after wound production, neutrophils and monocytes rapidly migrate to the wound [55]. In addition, inflammatory cells release lysosomal enzymes, reactive oxygen species, and clean cell debris. Subsequently, wound healing moves to proliferation through inflammation. In the proliferative phase, M2 macrophages induce anti-inflammatory cytokines, such as IL-10, and then a decrease in inflammatory cells is observed. Wound healing includes the participation and coordination of growth factors and cytokines. FGF-7 is a growth factor that accelerates wound healing, and significantly stimulates the migration and proliferation of keratinocytes [20]. The expression of FGF-7 has been shown to be dramatically upregulated in full-thickness excisional wounds of mice and humans after skin injury [56]. It was also observed to increase the proliferation of basal keratinocytes and improve re-epithelialization [57,58]. Considering these results, we reasonably concluded that FGF-7 in 2-GHC-FD-8 leads to rapid wound development through the inflammatory phase, thereby accelerating wound healing.

## 4. Conclusions

Our study investigated the in vivo wound healing effect of FDs based on gelatin, HA, and CMCS containing FGF-7. 2-GHC-FD-8 composed of 2% (*w*/*v*) gelatin, 20% HA (*w*/*w*), and 50% (*w*/*w*) CMCS showed improved cell proliferation due to its appropriate interconnected porosity, tensile strength, and elongation. In addition, 2-GHC-FD-8/F3 containing 8.0 × 10^−11^ M of FGF-7 enhanced routine wound healing by inducing positive histological changes and collagen formation, which was evaluated by H&E and MT staining. Gelatin, HA, CMCS, and FGF-7 have been investigated for wound healing; however, to our knowledge, this study is the first to evaluate wound healing in vivo. Although this research reports the potential of 2-GHC-FD-8-F3 on wound healing acceleration, further experiments need to be conducted, such as, FGF-7 activity, antibacterial activity, and extension of animal model for precise evaluation. In addition, larger sizes of wounds are recommended to evaluate the wound heling effect of 2-GHC-FD-8-F3.

## Figures and Tables

**Table 1:** Amounts of gelatin, HA, CMCS, and DMT-MM used for hydrogelation. For hydrogelation, Specific concentrations of gelatin (1, 2, 5, and 10 *w*/*v*%), HA (1, 5, and 10 *w*/*w*%), and CMCS (10, 50, and 100 *w*/*w*%) were used. As a condensation agent, 2 *w*/*v*% of DMT-MM was used.

FDs		Gelatin (*w*/*v*%)	HA (*w*/*w*%)	CMCS (*w*/*w*%)	DMT-MM (*w*/*v*%)	Hydrogelation
**1-**	**GHC-1**	1	1	10	2	×
	**GHC-2**			50		
	**GHC-3**			100		
	**GHC-4**		5	10		
	**GHC-5**			50		
	**GHC-6**			100		
	**GHC-7**		10	10		
	**GHC-8**			50		
	**GHC-9**			100		
**2-**	**GHC-1**	2	1	10	2	O
	**GHC-2**			50		
	**GHC-3**			100		
	**GHC-4**		5	10		
	**GHC-5**			50		
	**GHC-6**			100		
	**GHC-7**		10	10		
	**GHC-8**			50		
	**GHC-9**			100		
**5-**	**GHC-1**	5	1	10	2	O
	**GHC-2**			50		
	**GHC-3**			100		
	**GHC-4**		5	10		
	**GHC-5**			50		
	**GHC-6**			100		
	**GHC-7**		10	10		
	**GHC-8**			50		
	**GHC-9**			100		
**10-**	**GHC-1**	10	1	10	2	O
	**GHC-2**			50		
	**GHC-3**			100		
	**GHC-4**		5	10		
	**GHC-5**			50		
	**GHC-6**			100		
	**GHC-7**		10	10		
	**GHC-8**			50		
	**GHC-9**			100		

**Table 2:** Intradermal reactivity score evaluated by the degree of formation of erythema and callus, and edema.

**Formation of erythema and callus**	
No erythema	0
Slight erythema	1
Well-defined erythema	2
Intermediate erythema	3
Severe erythema (dark red) and callus	4
**Formation of edema**	
No edema	0
Slight edema	1
Well-defined edema	2
Intermediate edema (about 1 mm)	3
Severe edema (>1 mm)	4
Stimulus score at maximum	8

**Table 3:** Sample lists of wound dressing FDs, containing various concentrations of FGF-7, ranging from 2 to 16 × 10^−11^ M. the FD was prepared by mixing 2 *w*/*v*% of gelatin, 5 *w*/*w*% of HA, and 10 *w*/*w*% of CMCS.

Samples	Gelatin (*w*/*v*%)	HA (*w*/*w*%)	CMCS (*w*/*w*%)	FGF-7 (×10^−11^ M)
2-GHC-FD-8/F1	2	5	10	2
2-GHC-FD-8/F2				4
2-GHC-FD-8/F3				8
2-GHC-FD-8/F4				16
2-GHC-FD-8/F5				32
2-GHC-FD-8/F6				64

**Table 4:** Score of animal intradermal reactivity of 2-GHC-FD-1, 2-GHC-FD-4, and 2-GHC-FD-8 observed at 24, 48, and 72 h after injection using polar and non-polar solvents. The reactivity was confirmed by erythema, callus, and edema.

	Time	Control		2-GHC-FD-1,4,8	
		Polar Solvent	Non-Polar Solvent	Polar Solvent	Non-Polar Solvent
Erythema/Callus	24	0	0	0	0
	48	0	0	0	0
	72	0	0	0	0
Total score		0	0	0	0
Edema	24	0	0	0	0
	48	0	0	0	0
	72	0	0	0	0
Total score		0	0	0	0

## Data Availability

The data in this study are available on request from the cor- responding author.
